# Health-related quality of life in patients with liver cirrhosis following adjunctive nurse-based care versus standard medical care: a pragmatic, multicentre, randomised controlled study

**DOI:** 10.1136/bmjgast-2024-001694

**Published:** 2025-01-31

**Authors:** Maria Hjorth, Daniel Sjöberg, Anncarin Svanberg, Riccardo Lo Martire, Elenor Kaminsky, Fredrik Rorsman

**Affiliations:** 1 Centre for Clinical Research, Uppsala University, Falun, Sweden; 2 Department of Public Health and Caring Sciences, Uppsala University, Uppsala, Sweden; 3 Department of Medical Sciences, Uppsala University, Uppsala, Sweden; 4 School of Health and Welfare, Dalarna University, Falun, Sweden

**Keywords:** LIVER CIRRHOSIS, CLINICAL TRIALS, QUALITY OF LIFE

## Abstract

**Objectives:**

Patients have difficulties in understanding how to manage their liver cirrhosis. This highlights a need for support in comprehending health-related information, which remains largely lacking within liver cirrhosis care. Involvement of registered nurses (RNs) in outpatient liver cirrhosis care has potential to improve quality of care and reduce patient mortality. However, the benefits of nursing care on patients’ health-related quality of life (HRQoL) are scarcely studied. This study compared HRQoL in patients receiving either standard medical outpatient care or adjunctive, nurse-led care. The risk of malnutrition, decompensation events and mortality were also compared between the two study groups.

**Methods:**

This was a pragmatic, multicentre, randomised trial, which enrolled 167 patients with liver cirrhosis. The primary outcome measure, HRQoL, was assessed using the RAND-36 questionnaire. The physical component summary (PCS) and the mental component summary (MCS) scores of RAND-36 were compared, using linear mixed-effects models for repeated measures, at 12 and 24 months.

**Results:**

83 patients received standard medical care, and 84 patients received adjunctive, nurse-led care for 24 months. Due to unforeseen circumstances, the final study population of 167 participants was less than the intended 500. Group comparisons were non-significant of the PCS and MCS scores (−1.1, p=0.53 and −0.7, p=0.67, respectively), malnutrition (p=0.62) and decompensation events (p*=*0.46), after 24 months. However, mortality was three times higher in the control group compared with the intervention group (12 vs 4, p=0.04) after 24 months.

**Conclusions:**

In this study, adjunctive nurse-led care was not superior to standard medical outpatient care regarding HRQoL, risk of developing malnutrition or decompensation. However, RN involvement contributed to early identification of decompensation and reduced mortality.

**Trial registration number:**

NCT02957253.

**WHAT IS ALREADY KNOWN ON THIS TOPIC**

**WHAT THIS STUDY ADDS**

- Adjunctive nurse-led care did not have an impact on patients’ perceived HRQoL, risk of malnutrition or liver cirrhosis decompensation compared with standard medical care. Conversely, adjunctive nurse-led care may reduce mortality and contribute to early identification of decompensation events.

**HOW THIS STUDY MIGHT AFFECT RESEARCH, PRACTICE OR POLICY**

- HRQoL, measured with the generic RAND-36 instrument, could not identify any differences in outcomes among the 167 included patients. Therefore, other outcomes, such as patient-perceived quality of care and health economics, are recommended in future nursing intervention studies.

## Introduction

 Health-related quality of life (HRQoL) involves a person’s subjective experience of the physical, psychological and social aspects of their daily life. HRQoL is influenced by cultural context as well as personal values, goals and levels of independence.^1^ Compared with the general population, HRQoL is usually impaired in individuals with liver cirrhosis,^2^,^4^ the final stage of chronic liver disease.^5 6^ The transition from compensated liver cirrhosis to the decompensated phase is characterised by the development of severe symptoms, such as ascites, overt hepatic encephalopathy and/or bleeding from gastro-oesophageal varices.^5^ The decompensated phase is also afflicted with an increased risk of malnutrition,^7^ sarcopenia and physical frailty.^8^ The disease entails a mental burden, including depression,^9^ stigmatisation,^10 11^ anxiety^9 11^ and existential concerns.^11^ Accordingly, liver cirrhosis disease affects both the physical^34 12^,^14^ and psychological domains of HRQoL,^312^,^14^ making patients’ daily lives vulnerable and unpredictable.^11 15^ Patients with impaired HRQoL face a higher risk of hospitalisation related to liver cirrhosis.^2^ Therefore, identifying worsened HRQoL has a prognostic value in liver cirrhosis.^16^

It has been reported that regular registered nurse (RN)-led visits may add a holistic and person-centred perspective to the standard medical outpatient care,^17 18^ enabling patients to make informed decisions and undertake self-care actions to improve their health.^17 19 20^ These RN-led visits also have the potential to reduce mortality^21^ and increase patient-perceived quality of care.^20^ Despite patients repeatedly reporting difficulties in understanding how to manage their liver disease,^22^,^26^ support from healthcare providers that facilitate access to and understanding of health-related information^27^ remains largely insufficient in outpatient care. Hence, continuous and person-centred collaboration with healthcare providers,^28^ delivered by RNs within hepatology healthcare teams, may improve patients’ HRQoL, as has been confirmed in the management of other somatic diseases.^29^ However, only a few studies have previously evaluated the effectiveness of RN involvement in improving HRQoL for patients with liver cirrhosis.^30 31^ None of these applied to both compensated and decompensated phases of the disease. Accordingly, the primary aim of this study was to compare HRQoL in patients with either decompensated or compensated liver cirrhosis after receiving either standard medical outpatient care or an adjunctive person-centred nurse-led intervention, the Quality Liver Nursing Care Model (QLiNCaM).^20 32^ Secondary aims were to compare the risk of malnutrition, decompensation events and mortality rates between the two study groups.

## Methods

### Design

This study had an experimental comparative design and was carried out as a pragmatic, multicentre, randomised parallel-group trial. Comparisons were made on the primary outcome measure, HRQoL and the secondary outcome measures—risk of malnutrition, decompensation and mortality, over a 24-month period of standard medical care versus an adjunctive person-centred nurse-led intervention.

We hypothesised that perceived HRQoL, measured with the RAND-36,^33 34^ would increase in the study group receiving the adjunctive person-centred nurse-led intervention compared with those receiving standard medical care. The a priori sample size was estimated at 500 participants, based on the primary outcome measure, RAND-36, having two summary scores: the physical component summary (PCS) and the mental component summary (MCS).^33 34^ To ensure a power of 80%, a Bonferroni-corrected alpha of 2.5%, an SD of 9 and an estimated treatment difference of 2.5 points between the groups resulted in a goal of 250 participants per study group.

The study adhered to the Consolidated Standards of Reporting Studies guidelines^35^ (online supplemental table 1). Full details of the study design, methodology and a priori statistical analysis plan were reported in advance (ClinicalTrials.gov NCT02957253). The intervention and its intended outcomes were described in detail in a study protocol,^32^ and results from the secondary outcome, patient-perceived quality of care, have been reported previously.^20^

### Setting and patients

Eligible patients were aged 18–85, had been diagnosed with liver cirrhosis within the previous 24 months and were scheduled to receive standard medical care at one of the six Swedish hepatology outpatient clinics, comprising two county hospitals and four university hospitals. Patients with severe comorbidities, persistent hepatic encephalopathy or those who were non-Swedish speaking were excluded. On obtaining written consent, participants were consecutively allocated in blocks of 4, 6 or 8 and stratified by study centre and disease severity (compensated *vs* decompensated). A concealed, computerised randomisation sequence (Randomise.Net, Interrand, Ottawa, Canada) was used. The allocation ratio was 1:1 to either the intervention group or the control group. Recruitment took place from 2016 to 2020, with an individual time for study participation of 24 months (±2 months). Data were collected at 12 and 24 months of follow-up by RNs assigned specifically to each study group.^32^

### Study groups

The control group received care according to the current medical guidelines for outpatient management of liver cirrhosis. Standard medical care included telephone calls to RNs as needed, outpatient visits and telephone contact with physicians, a screening programme for hepatocellular carcinoma, endoscopy for variceal control and laparocentesis for ascites management. Neither outpatient visit intervals nor inpatient care were affected by the study.

**Figure 1:**
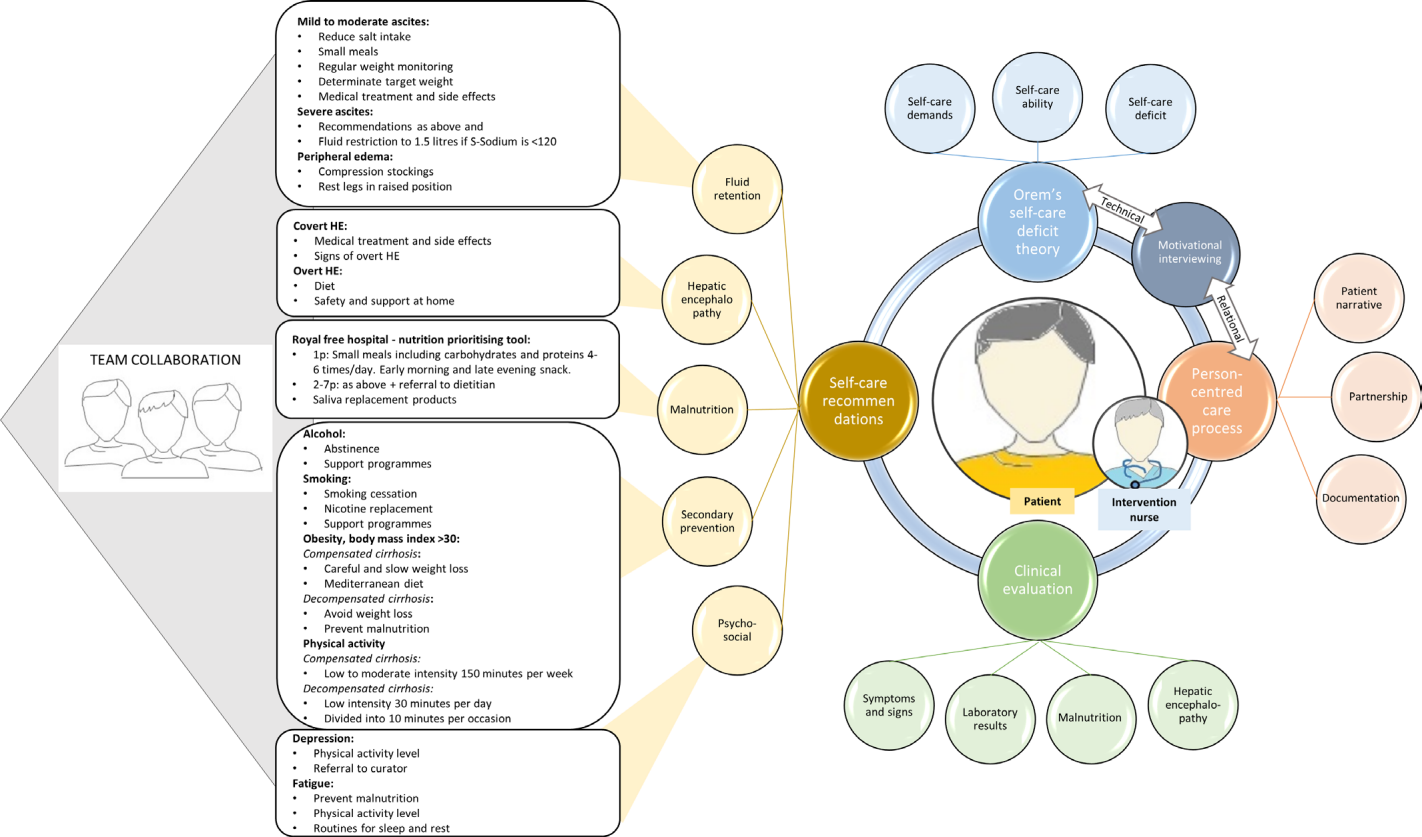
Description of the content of the adjunctive person-centred nurse-led care intervention, QLiNCaM The illustration is reproduced from a previous publication^20^ under a Creative Commons licence. QLiNCaM, Quality Liver Nursing Care Model.

In the intervention group, QLiNCaM was added to standard medical care, based on Orem’s nursing theory^36^ and person-centred care.^37^ The intervention was delivered by RNs educated in the intervention, and the person-centred communication technique used was motivational interviewing.^38^ The patient’s narrative, together with the RN’s assessment of signs and symptoms, formed the content of each visit, with a focus on self-care to prevent disease progression. If necessary, the RN could involve other team members, for example, physician or dietitian, in the patient’s care (figure 1). Visit frequency was individualised but framed with recommendations for closer follow-up for patients with decompensated disease.^20 32^

### Outcome measures and data collection

The primary outcome measure, HRQoL, was assessed using the Swedish version^39^ of the original RAND-36 questionnaire^34^ via touchscreen devices. RAND-36, the free version of Short-Form-36,^40^ provides insights into patient’s physical and mental health, which may be compared with the general population and other disease populations. RAND-36 has been used repeatedly in liver cirrhosis populations.^3 4 12^ The questionnaire includes 36 category scale items, each with response alternatives between 2 and 6. Each response was converted to a scale from 0 to 100, with higher values predicting better health. From the 36 items, 8 subscales were derived: (1) physical functioning, (2) role limitations caused by physical health problems, (3) pain, (4) energy/fatigue, (5) social functioning, (6) role limitations caused by emotional problems, (7) emotional well-being and (8) general health perception. Based on the eight subscales, we calculated the two summary components: the PCS and the MCS^33 34^ under the assumption that these were correlated,^41^ using weight scores derived from a mid-Swedish population as a reference.^42^

The secondary outcome measure ‘risk of malnutrition’ was assessed using the Royal Free Hospital–Nutritional Prioritising Tool.^16^ This score was calculated by the RNs based on each patient’s history of alcohol hepatitis, fluid retention, unplanned weight loss, dietary intake and body mass index. The Royal Free Hospital–Nutritional Prioritising Tool correlates with the deterioration of cirrhosis and classifies participants into low (0 points), medium (1 point) or high (2–7 points) risk categories for malnutrition.

Data on decompensation events, that is, occurrence of ascites, oesophageal variceal bleeding or overt hepatic encephalopathy, were collected from medical records at 12 and 24 months.

Due to the poor prognosis with high mortality rates, especially in the decompensated liver cirrhosis phase, we decided to also investigate the possible effect on mortality between the study groups. This decision was made after the study protocol^32^ was published. Mortality data were extracted from medical records for all patients completing the study. For participants who had withdrawn, data were retrieved from the Swedish population registry.

The outcome measures for the control group and intervention group were collected at each of the six participating sites. RNs dedicated to either the control group or intervention group were responsible for data collection during visits to the hepatology outpatient clinic at baseline, 12 months and 24 months. Covert hepatic encephalopathy was confirmed with two independent psychometric tests: the psychometric hepatic encephalopathy score^43^ and continuous reaction time.^44^ Health literacy was assessed with the Newest Vital Sign.^45^

### Data analysis

Data were analysed according to the intention-to-treat principle, where participants were coded to their allocation status. In line with the a priori statistical analysis plan (ClinicalTrials.gov NCT02957253) and the study protocol,^32^ two separate linear mixed-effects models for repeated measures were used to compare the RAND-36 PCS and MCS scores^33^ between the intervention and control groups at each time point (nlme V.3.1-162 in R V.4.2.3). The models included fixed factors for time (baseline, 12 months and 24 months), treatment (intervention or control), their interaction and the baseline disease status (decompensated or compensated). Recruiting hospital was included as a random effect. Temporal dependency per patient was managed with an unstructured covariance matrix for the repeated measures. Based on these models, marginal means were estimated per time point and intervention group, as well as globally across time points (marginal effects V.0.15.1 in R V.4.2.3). The primary endpoint was set at 24 months after baseline. Alpha was corrected for the endpoint primary outcome measure (i.e., alpha <0.05/2). Analyses at 12 months were not adjusted for multiplicity.

Comparison between the two study groups for the secondary outcome measure, the Royal Free Hospital–Nutritional Prioritising Tool score (0–7 points), from baseline to 12 months and 24 months, respectively, was analysed with Mann-Whitney U test. Decompensation events were compared between the study groups with Pearson’s χ^2^ test, and mortality with Fisher’s exact test. An alpha value of <0.05 was regarded to be significant.^46^

## Results

### Patients’ characteristics

**Figure 2:**
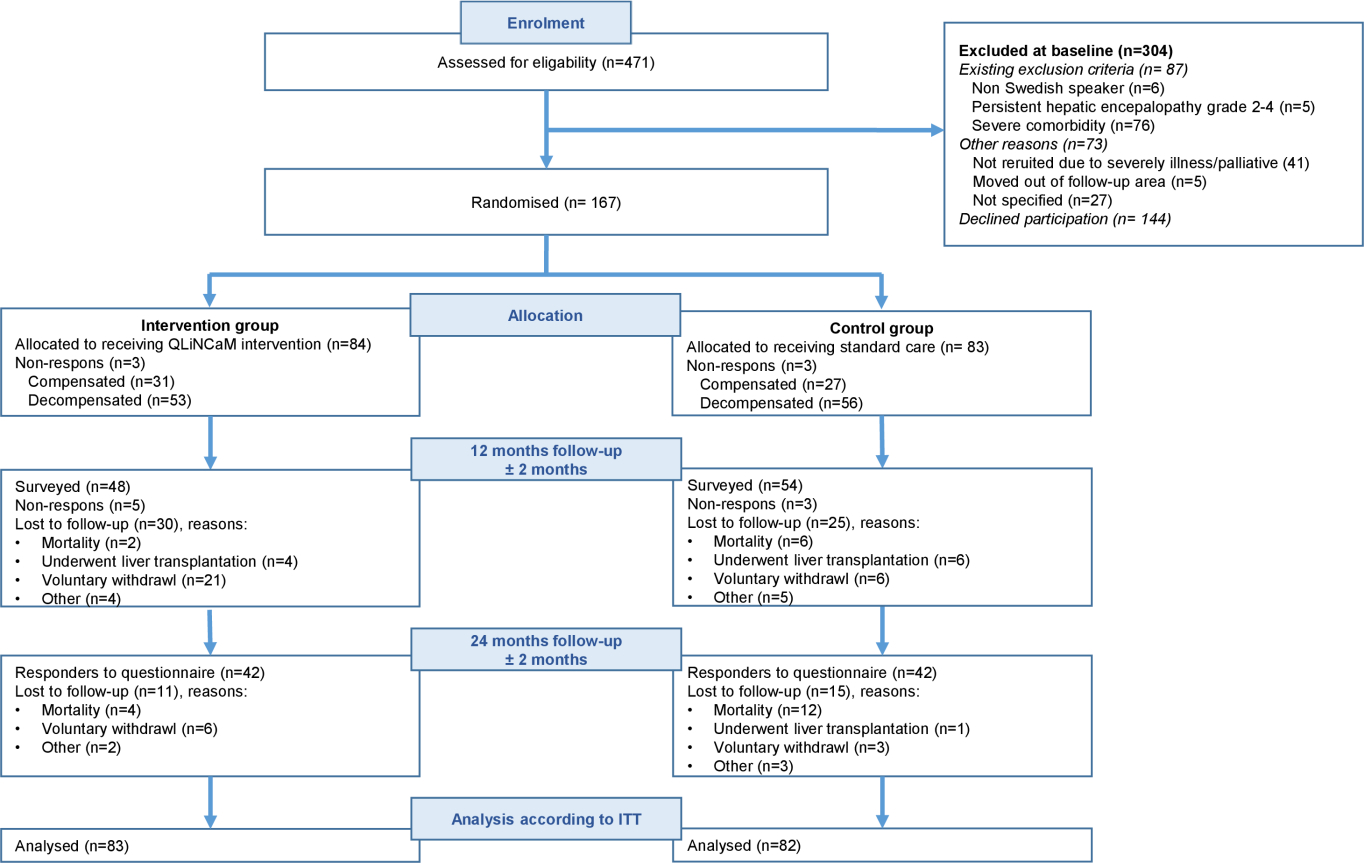
CONSORT diagram of the enrolment process and allocation of participants. Two participants did not answer the questionnaires and were not included in the intention-to-treat (ITT) analysis. The illustration is reworked based on the primary aim of the study, reproduced from a previous publication.^20^

Of the 384 eligible patients, 167 were enrolled in the two study groups: the control group (n=83) and the intervention group (n=84) (figure 2). In total, 109 patients had previous experience with liver cirrhosis decompensation. At baseline, the most frequently reported symptoms were fatigue (control group n=47; intervention group n=40), swollen legs (control group n=26; intervention group n=26), muscle weakness (control group n=21; intervention group n=26) and reduced libido or potency (control group n=24; intervention group n=17). During the study, the proportion of patients with severe disease, as measured by the Child Pugh, decreased, mainly caused by loss to follow-up (figure 2). For example, at baseline, 41% of participants were classified as Child Pugh B or C, but by 24 months, the corresponding figure was 35%. The most common diagnosis was alcohol-related liver disease. At baseline, 25 of the 86 patients with alcohol-related liver disease presented positive B-phosphatidylethanol (B-Peth)^47^ in blood (≥0.05 µmol/L). Five of the 25 patients who consumed alcohol at baseline refrained alcohol intake during the 24 months study time. 10 of the patients with alcohol-related liver disease, having B-Peth≤0.05 umol/L at baseline, relapsed to alcohol consumption. At 24 months, data were missing for 33 participants with alcohol-related liver disease. Baseline characteristics are presented in table 1. The number of visits to physicians each year remained equal in the two study groups (mean=1.3). In addition, the intervention group received the adjunctive person-centred nurse-led intervention a mean of 2.6 times in the first 12 months, and a mean of 2.8 times in the last 12 months of the study.

**Table 1:** Characteristics of participating patients

	Intervention group(N=84)n (%)	Control group(N=83)n (%)
Female gender	37 (44)	35 (42)
Age		
18–64	42 (50)	41 (50)
65–85	42 (50)	42 (50)
Country of birth		
Sweden	78 (93)	76 (91)
Europe	5 (6)	3 (4)
Other	1 (1)	4 (5)
Marital status		
Single	32 (38)	27 (33)
Cohabiting	52 (62)	55 (67)
Missing	0 (0)	1 (1)
Education		
Elementary school	23 (27)	19 (23)
Upper secondary school	41 (49)	41 (49)
University	20 (24)	23 (28)
Liver cirrhosis diagnosis		
Alcohol related liver disease	48 (57)	38 (46)
Autoimmune hepatitis	6 (7)	7 (8)
Viral hepatitis C	4 (5)	3 (4)
Metabolic dysfunction-associated steatotic liver disease	14 (17)	8 (9)
Cryptogenic	9 (11)	20 (25)
Primary biliary cholangitis	2 (2)	3 (4)
Other*	1 (1)	4 (5)
Covert hepatic encephalopathy	23 (27)	23 (28)
Missing	6 (7)	7 (8)
Previous liver cirrhosis decompensation†	53 (63)	56 (67)
Ascites	36 (43)	43 (52)
Overt hepatic encephalopathy	14 (17)	7 (8)
Episode of variceal bleed	17 (20)	19 (23)
Child Pugh score		
A	52 (62)	47 (57)
B	26 (31)	31 (37)
C	6 (7)	5 (6)
Health literacy		
Adequate	32 (39)	37 (45)
Possible limited	50 (61)	46 (55)
Missing	2 (2)	0 (0)
Comorbidity‡		
None	50 (60)	49 (59)
1–2	34 (40)	30 (36)
>2	0 (0)	4 (5)

* Liver cirrhosis due to toxicity, congestive heart disease, or α−1 antitrypsinaemia.

† Presentation of ascites, overt hepatic encephalopathy or variceal bleeding before enrolment.

‡ cComorbidity: diabetes, heart disease, inflammatory bowel disease, allergy, depression, arthrosis, chronic obstructive pulmonary disease, rheumatism, and undergone cancer.

### Effectiveness of the QLiNCaM intervention on patients’ perceived HRQoL

We found no significant difference between the PCS and MCS scores of RAND-36 in patients who received the adjunctive person-centred nurse-led intervention compared with those receiving standard medical care. At 12 months, the difference on the 100-point RAND-36 scale was −1.7 for PCS and 0.3 for MCS in the intervention group compared with the control group. After 24 months, the difference was −1.1 for PCS and −0.7 for MCS (table 2).

**Table 2:** Comparison of RAND-36 physical and mental component summary (PCS and MCS) scores expressed in estimated marginal means with 95% CI at 12 and 24 months between the two study groups

Outcome	Intervention group		Control group		Difference(95% CI)	P value
	Patients; n	Point estimates (95% CI)	Patients; n	Point estimates (95% CI)		
Baseline	81		80			
PCS		44.1 (42.4 to 45.8)		44.1 (42.4 to 45.8)		
MCS		46.9 (45.5 to 48.3)		46.9 (45.5 to 48.3)		
12 months	52		55			
PCS		44.9 (42.3 to 47.5)		46.7 (44.1 to 49.2)	−1.7 (−4.8 to 1.4)	0.28
MCS		48.7 (46.4 to 41.0)		48.4 (46.2 to 50.7)	0.3 (−2.7 to 3.2)	0.85
24 months	48		40			
PCS		44.6 (42.0 to 47.2)		45.7 (42.8 to 48.5)	−1.1 (−4.5 to 2.3)	0.53
MCS		47.6 (45.4 to 49.9)		48.3 (45.8 to 50.8)	−0.7 (−3.8 to 2.5)	0.67

In the eight subscales of RAND-36, both study groups remained similar. A group comparison of all subscales per time point is visually presented in online supplemental figure 1.

### Secondary outcome measures

The Mann-Whitney U test revealed no significant differences when comparing the risk of malnutrition, as measured by the Royal Free Hospital–Nutritional Prioritising Tool, between the two groups (p=0.33 at 12-month follow-up; p=0.62 at 24-month follow-up). Missing data for the Royal Free Hospital–Nutritional Prioritising Tool were n=29 and n=42 for the control group, and n=30 and n=39 for the intervention group, at 12 and 24 months, respectively (table 3).

**Table 3:** Results of secondary outcome measure analyses at 12 and 24 months between the two study groups

Data source	Outcome measure	12 months			24 months		
		Intervention groupN	Control groupN	P value for group comparison	Intervention groupN	Control groupN	P value for group comparison
Royal Free Hospital–Nutritional Prioritising Tool	Risk of malnutrition			0.33			0.62
	Low risk	38	44		32	26	
	Medium risk	13	7		12	13	
	High risk	3	3		1	2	
	Unknown/missing	30	29		39	42	
Medical journals	Decompensation events			0.25			0.46
	Occurrence of ascites, oesophageal variceal bleeding or overt hepatic encephalopathy	11	7		7	9	
	No event of decompensation	46	53		43	37	
	Unknown/missing	27	23		34	37	
Medical journals and Swedish population registry data	Mortality			0.17			0.04
	Dead	2	6		4	12	
	Alive	82	77		80	71	

The mean time from randomisation to the first or subsequent observed decompensation event was 455 days in the control group, compared with 343 days in the intervention group. During the 24-month study period, 12 patients in the control group and 17 patients in the intervention group experienced one or more episodes of liver cirrhosis decompensation. The Pearson’s χ^2^ test showed no significant differences in decompensation events between the two study groups. Missing data for liver cirrhosis decompensation were n=37 for the control group and n=34 for the intervention group (table 3).

A total of 8 and 16 patients died before the 12-month and 24-month follow-ups, respectively. No statistically significant difference in mortality was observed at 12 months (p=0.17*,* n=2 *vs* n=6). However, at 24 months, the Fisher’s exact test indicated that mortality was lower (p=0.04, n=4 *vs* n=12) in the intervention group compared with the control group (table 3). The comparison of mortality between the two study groups was not part of the a priori study protocol. Instead, this analysis was part of the missing data analysis. The difference in mortality rates is, hence, a secondary finding with full data coverage.

During the participant recruitment period from 2016 to 2020, new national guidelines were published, recommending RN involvement as a standard practice in liver cirrhosis outpatient care in Sweden. This prevented further recruitment of participants for the study.

## Discussion

This 24-month intervention study primarily aimed to compare HRQoL in patients receiving either medically based liver cirrhosis outpatient care or an adjunctive person-centred nurse-led intervention. The study involved six Swedish hospitals and provided longitudinal data following a nurse-led liver cirrhosis outpatient intervention, which is one of the most comprehensive of its kind.^21 48^ Our ambition was to prevent disease deterioration in the compensated liver cirrhosis group, which was in line with the new paradigm of liver cirrhosis care.^49^ Theoretically, this approach could also improve patients’ HRQoL. However, in contrast to an improved patient-perceived quality of care from the QLiNCaM intervention at 12 months that we previously reported (21), we found no significant difference in HRQoL, as measured by the RAND-36 composite scores, PCS and MCS^33^ (table 2), between the two study groups. Our non-superior results are consistent with previous reports on the effectiveness of RN-led interventions in liver cirrhosis on patients’ HRQL,^30 31^ although in opposition to RN involvement in the management of other somatic diseases.^29^ In contrast to what one might expect in cirrhosis, which is a progressive and deteriorating disease,^2 14^ we found that both the reported PCS and MCS (table 2), as well as the Child Pugh score, slightly improved over the 24-month study period. We believe that this seemingly apparent improvement may, at least partially, be due to the loss of follow-up of patients with more severe disease and higher mortality rates in the control group, which may have obscured the effect of the RN-intervention in our study. One could also speculate that the liver cirrhosis itself, as well as a simultaneous alcohol use, impact patients’ HRQoL to such an extent that is unaffected by nursing interventions. Future studies should address other outcome measures, for example, readmissions,^18 21 31 50 51^ mortality rates,^18 21^ quality of care^20 52^ and health economics.^18^

Moreover, there was no evidence that the QLiNCaM intervention reduced the incidence of malnutrition or decompensation events (table 3). Nevertheless, in the intervention group, the mean time to identify decompensation events was 112 days shorter than in the control group, which probably reflects the RNs preventive role in identifying cirrhosis complications, consistent with recent publications of Kalo *et al*^50^ and Giles *et al*.^51^ Despite not being one of our predefined outcomes, interestingly enough, we found support for a two-thirds reduction in mortality in the intervention group compared with the control group (table 3). The lower mortality rates may be a result of the earlier identification of decompensation events in the intervention group. Hence, our findings support previous interventions studies^5051 53^,^55^ that nurse-involvement in liver cirrhosis care may decrease long-term mortality.

### Strengths and limitations

A key strength of this randomised controlled study is its pragmatic design, which facilitates the implementation of the QLiNCaM intervention in clinical settings. Another strength is that we recruited and stratified patients with both decompensated and compensated liver cirrhosis at the time of allocation. This approach differs from previous intervention studies that only included patients with decompensated disease.^18 30 31^

We aimed to recruit 500 participants but only identified 384 patients who met the eligibility criteria for randomisation (figure 2), which is a considerable limitation. Although we extended the period for inclusion of participants by 12 months, recruiting patients in clinical settings was challenging. Patients with the most severe liver disease were often reluctant to provide consent, which may impact the generalisability of the study’s results. However, as the representation of the patients’ background characteristics was equal in the two study groups, this is unlikely to influence the internal validity of the study. There was a larger-than-expected drop-out rate, and the COVID-19 pandemic hampered the completion of the intervention and data collection between 2020 and 2022. Moreover, the introduction of new national guidelines, emphasising the multidisciplinary nature of cirrhosis care, interfered with the study’s objectives, preventing further extension of the recruitment period. The under-recruitment and more patients than expected that were lost to follow-up may be systematic with risk for selection bias.

We hypothesised that patients’ HRQoL would be mediated by social contact with the RNs, as delivered in the QLiNCaM intervention. The fewer visits during the COVID-19 pandemic may have reduced the effect size of patients’ HRQoL between the two study groups. We also acknowledge the risk that missing data could be systematic, which complicated the interpretation of secondary outcome measures such as ‘risk of malnutrition’ and ‘decompensation events’. Therefore, we decided to include mortality in the comparative analyses, in addition to what was described in the statistical analysis plan, at ClinicalTrials.gov NCT02957253. The addition of mortality data should be considered a strength, as data covered all participants, including those who withdrew from participation, and provided additional insights into the potential impact of RN-led outpatient cirrhosis care on mortality. Due to a smaller sample size than planned, we refrained from conducting any subgroups analyses, which was in line with the a priori statistical analysis plan (ClinicalTrials.gov NCT02957253) and the study protocol.^32^

## Conclusions

We found no evidence that adjunctive nurse-led care is superior to standard medical care in improving HRQoL in patients with liver cirrhosis, nor was there any impact on malnutrition or decompensation events. However, this study suggests that RN involvement may contribute to the early detection of decompensation events, which has the potential of reducing mortality in patients with liver cirrhosis and improving patients’ satisfaction with healthcare, as previously reported.^20^ In future nurse-led intervention studies on liver cirrhosis, we recommend monitoring decompensation events as an important factor to study. Furthermore, we recommend examining other clinically relevant outcomes, such as person-centred care measures, healthcare needs, health economics or qualitative evaluations of patient experience.

## supplementary material

10.1136/bmjgast-2024-001694 online supplemental figure 1

10.1136/bmjgast-2024-001694 online supplemental file 1

## Data Availability

Data are available on reasonable request.
