# Supplementary figures and images for: Health-related quality of life in patients with liver cirrhosis following adjunctive nurse-based care versus standard medical care: a pragmatic, multicentre, randomised controlled study
Source: BMJ Open Gastroenterol. 2025 Jan 31;12(1):e001694. doi: 10.1136/bmjgast-2024-001694 (PMC11792282; doi:10.1136/bmjgast-2024-001694)

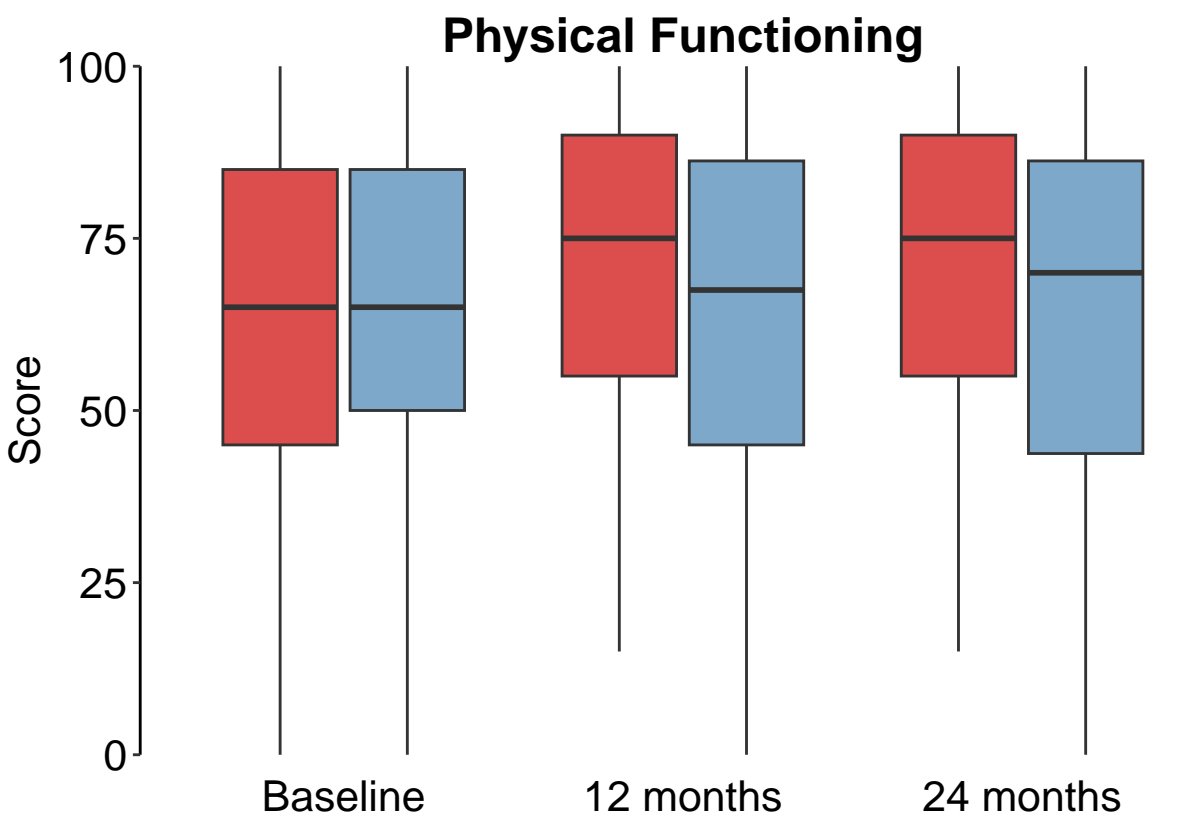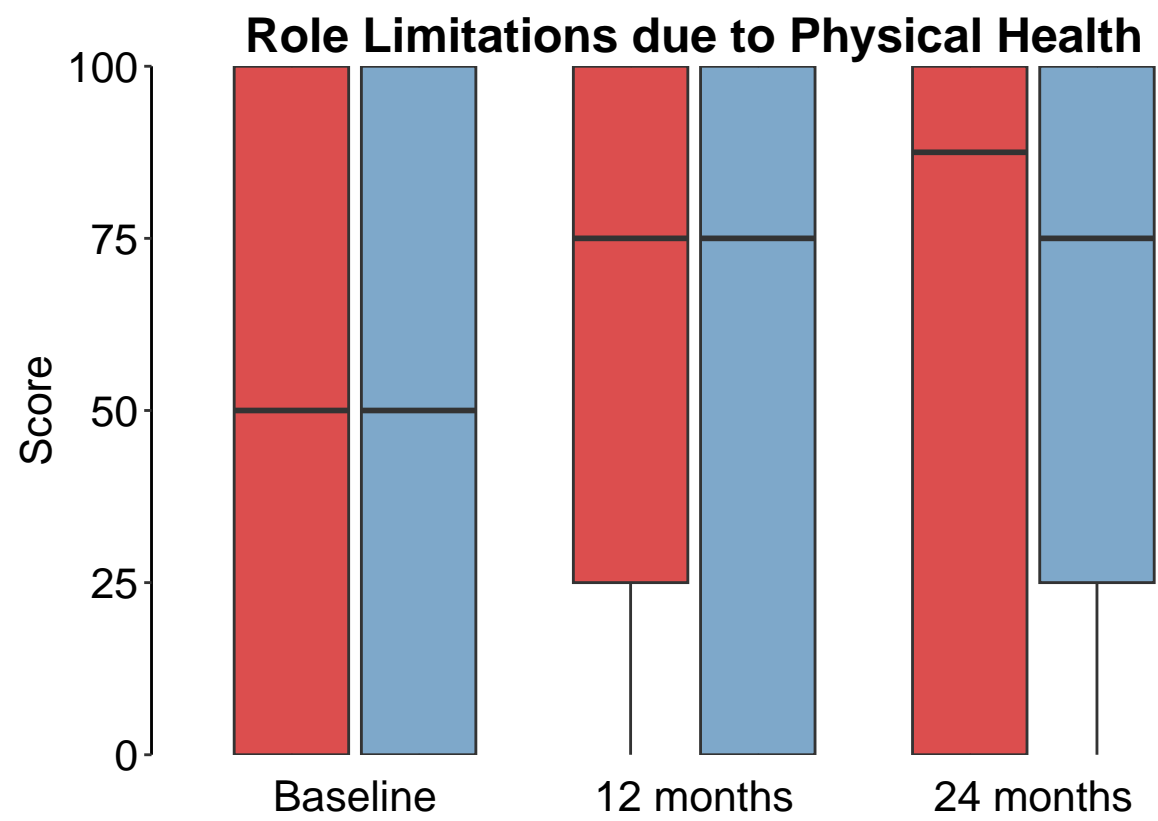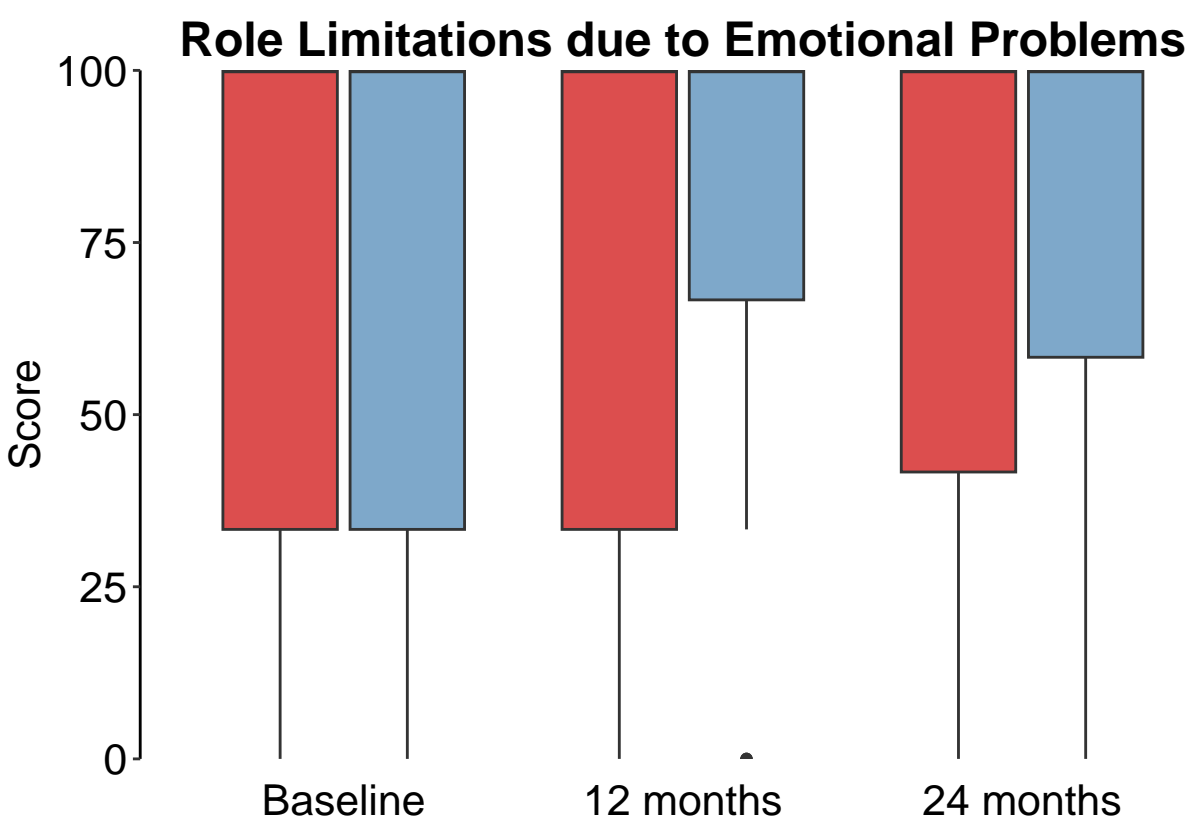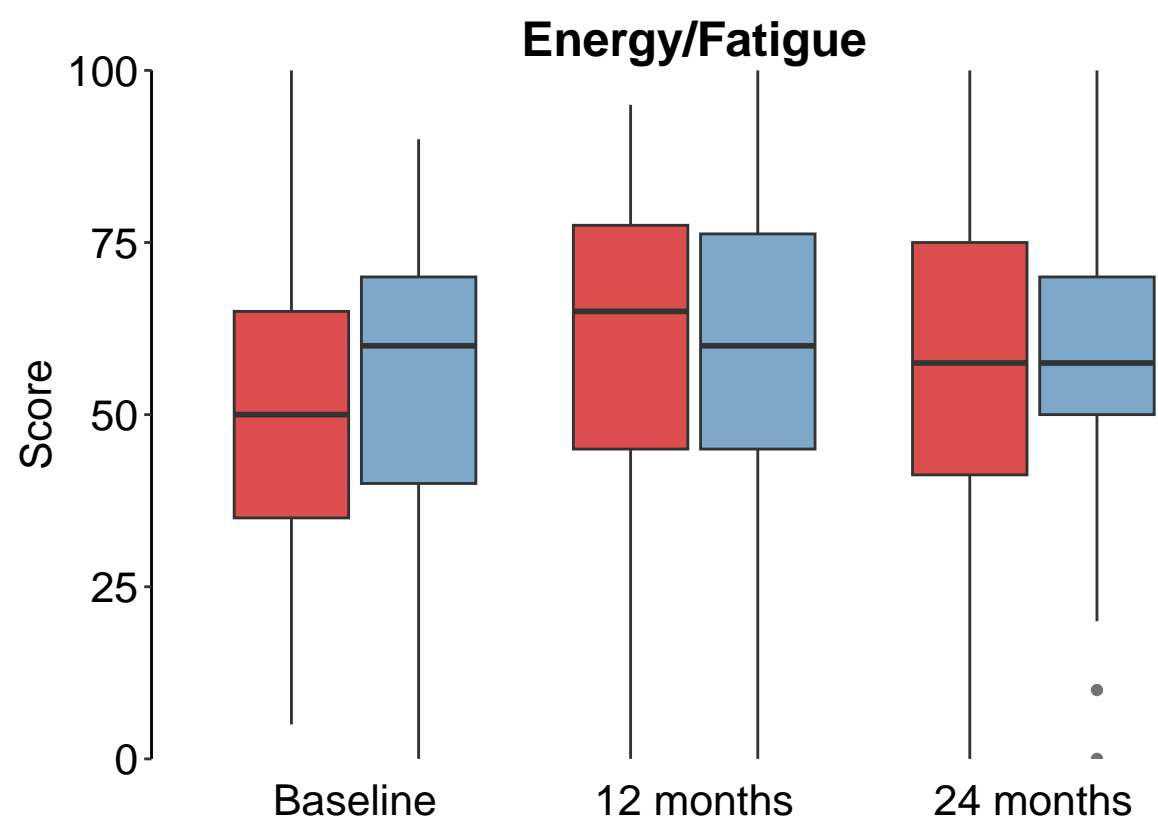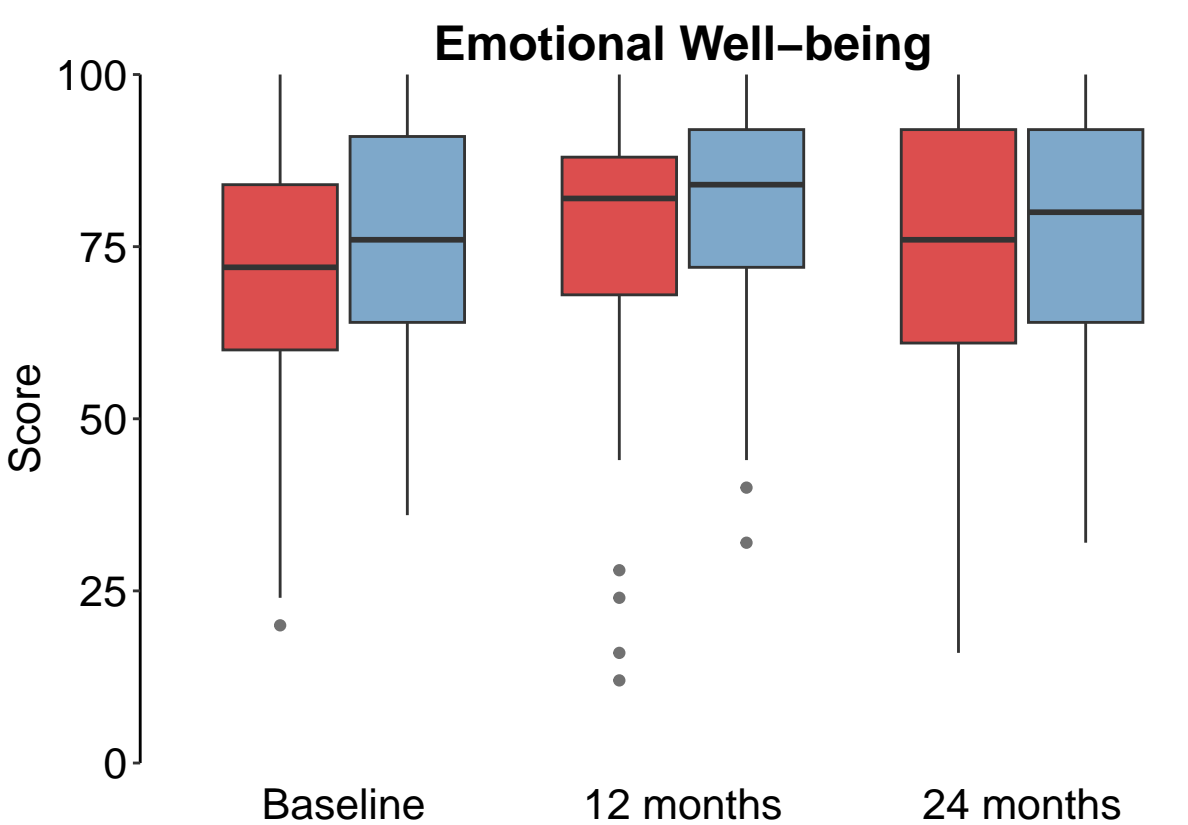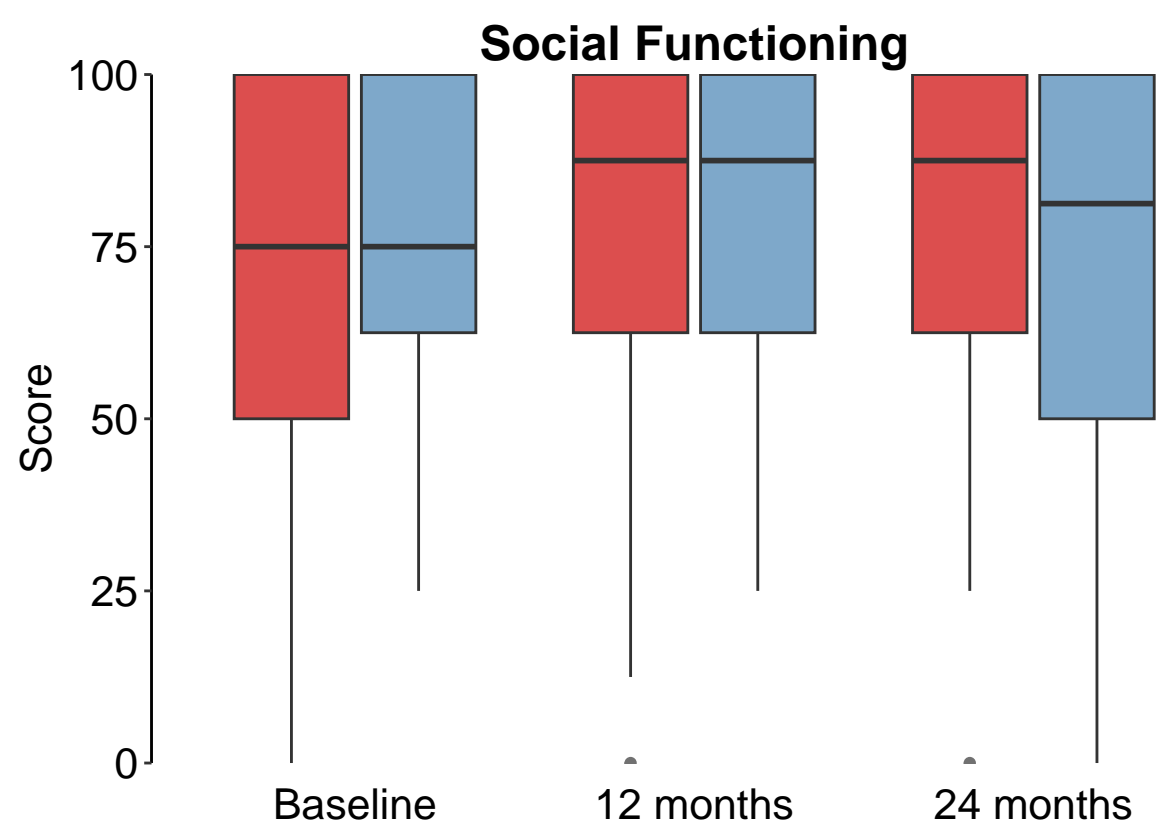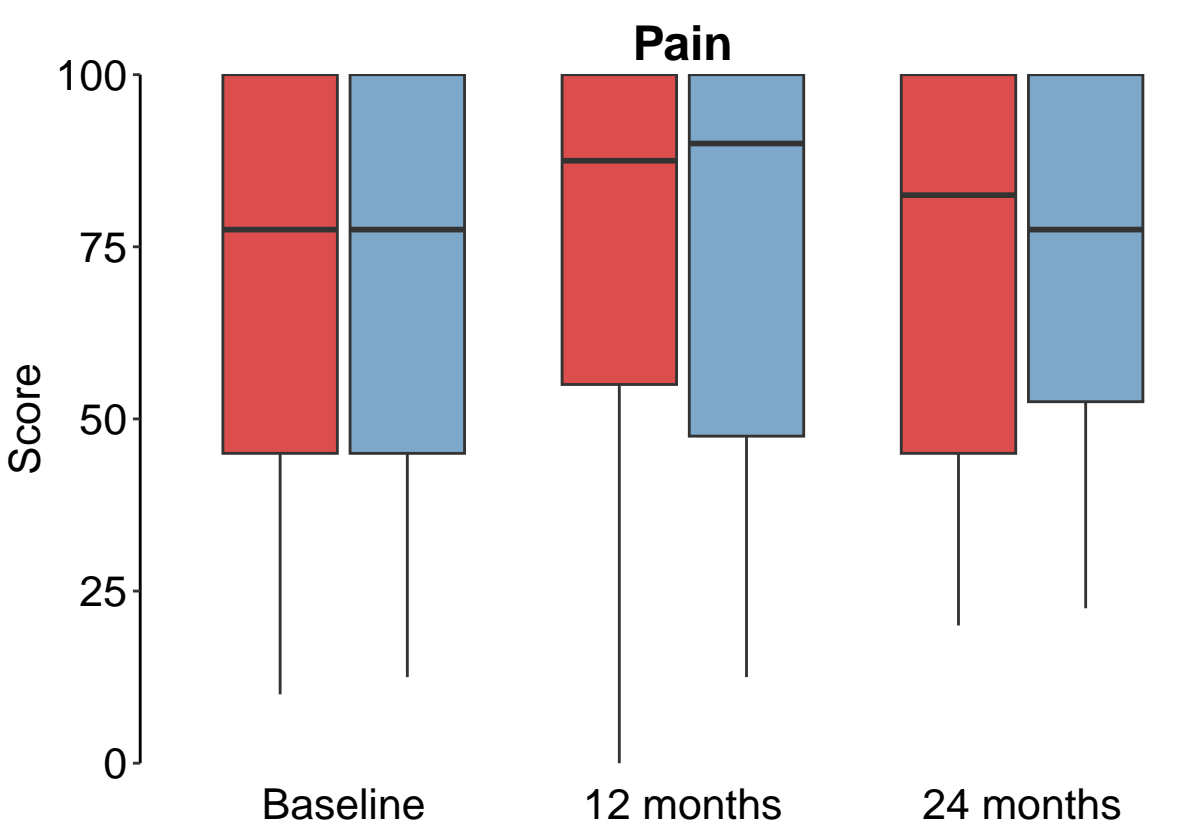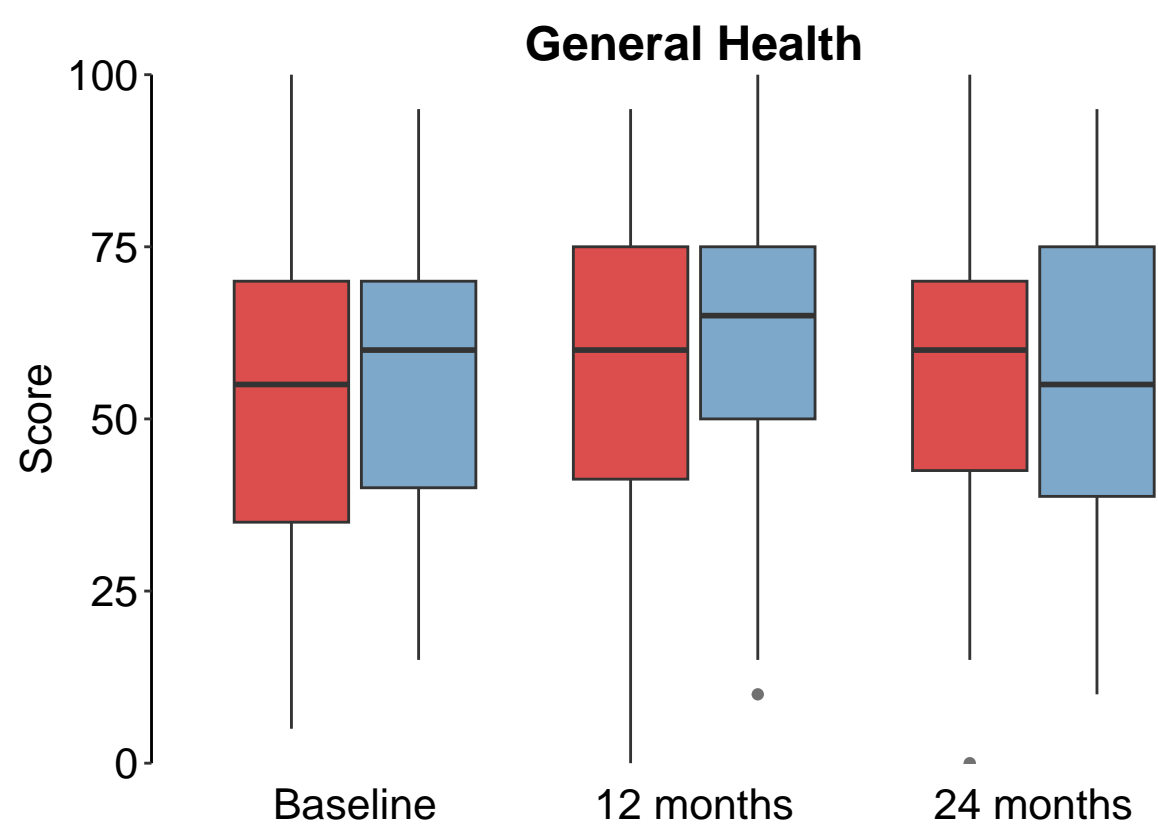

Supplement: online supplemental figure 1 [file bmjgast-12-1-s001.pdf]
